# Comparing population-level humoral and cellular immunity to SARS-Cov-2 in Bangalore, India

**DOI:** 10.1038/s41598-024-54922-z

**Published:** 2024-03-08

**Authors:** Anup Malani, Jayashree Aiyar, Andrea Sant, Neha Kamran, Manoj Mohanan, Saloni Taneja, Bartek Woda, Wanran Zhao, Anu Acharya

**Affiliations:** 1 University of Chicago, Chicago, IL USA; 2 Syngene International LTD, Bangalore, India; 3 Department of Microbiology and Immunology, University of Rochester Medical Center, Rochester, NY USA; 4 Sanford School of Public Policy, Duke University, Durham, NC USA; 5 University of Southern California, Los Angeles, CA USA; 6 Present Address: Amazon, Chicago, IL USA; 7 Mapmygenome India LTD, Hyderabad, India

**Keywords:** Viral infection, Epidemiology, Viral infection

## Abstract

Two types of immunity, humoral and cellular, offer protection against COVID. Humoral protection, contributed by circulating neutralizing antibodies, can provide immediate protection but decays more quickly than cellular immunity and can lose effectiveness in the face of mutation and drift in the SARS-CoV-2 spike protein. Therefore, population-level seroprevalence surveys used to estimate population-level immunity may underestimate the degree to which a population is protected against COVID. In early 2021, before India began its vaccination campaign, we tested for humoral and cellular immunity to SARS-Cov-2 in representative samples of slum and non-slum populations in Bangalore, India. We found that 29.7% of samples (unweighted) had IgG antibodies to the spike protein and 15.5% had neutralizing antibodies, but at up to 46% showed evidence of cellular immunity. We also find that prevalence of cellular immunity is significantly higher in slums than in non-slums. These findings suggest (1) that a significantly larger proportion of the population in Bangalore, India, had cellular immunity to SARS-CoV-2 than had humoral immunity, as measured by serological surveys, and (2) that low socio-economic status communities display higher frequency of cellular immunity, likely because of greater exposure to infection due to population density.

## Introduction

Infection with SARS-CoV-2 (COVID) triggers both humoral antibodies and cellular immunity that offers protection against health burdens from subsequent COVID infections^1–5^. For those individuals who were previously infected, circulating antibodies reactive to the receptor binding sites of the spike protein can provide immediate protection from infection. Protection via cellular immunity is often delayed^6,7^ and the effector functions associated with T cell immunity typically permit infection, but mitigate disease severity^8^. Cellular immunity and circulating antibodies decay over time^9^, but there is some evidence that cellular immunity persists longer than humoral immunity. Additionally, and probably most important, the mutations and accumulated drift in the SARS-CoV-2 spike protein (reviewed in^2,10,11^) often renders many antibodies ineffective for protection. The broader antigen specificity of CD4 and CD8 T cells permits SARS-CoV-2 reactivity in the face of spike genetic variation (reviewed in^12–17^).

Population-level surveys of immunity estimate how vulnerable a population is to an infectious disease. They are an important input into formulating policy on non-pharmaceutical interventions and allocation of scare vaccines^18,19^. With one exception^5^, population-level surveys of immunity to SARS-CoV-2 measure humoral immunity (seroprevalence). Because humoral immunity can decay more quickly than cellular immunity, these surveys may underestimate immune protection from future SAR-CoV-2 infection. The challenge with measuring cellular immunity compared to humoral immunity is that it requires a large blood volume, fewer labs are capable of testing for cellular immunity, and cellular immunity tests are more expensive, with fewer validated outcomes^20,21^.

We address two questions. First, to what extent do seroprevalence surveys underestimate immunity in the community? Second, what are the correlates of humoral and cellular immunity? In particular, does each type of immunity vary by socio-economic status and demographic group? Specifically, we measure seropositivity and cellular memory using blood specimens drawn in February 2021, just after India’s first COVID wave, but before India began scaling its SARS-Cov-2 vaccination program, from two populations in Bangalore, India. One sample was representative of populations living in slums and the other sample of populations in non-slums. We measure humoral immunity via enzyme-linked immunoassays (ELISA) for the SARS-Cov-2 spike protein and rapid tests for neutralizing antibodies to the receptor binding domain (RBD) of that spike protein. We measure cellular memory with a functional assay that measures the concentration of interferon-gamma (IFN*γ*) and interleukin-2 (IL-2) cytokine production from CD4+ and CD8+ T cells^17^, respectively, amongst peripheral blood mononuclear cells (PBMCs) exposed to peptides representing the translated sequence of the entire spike protein, including the receptor binding domain. We also survey all subjects regarding their demographics and socioeconomic conditions. We compare and investigate the relationships among post-COVID immunity by community type, humoral immunity status, and cellular immunity across age groups.

## Methods

### Approvals and informed consent

This study was approved by and conducted under the aegis of the Principal Scientific Advisor to the Prime Minister of India and the Government of Karnataka state. IRB approval was obtained from the Karesa Ethics Committee (Bangalore, India). (See Methods: Approvals in Supplement for details.) All methods were performed in accordance with the relevant guidelines and regulations set forth in our approvals.

### Timing

Recruitment and biospecimen sampling took place between January 10, 2021, and March 4, 2021. This was after India’s initial COVID wave (September 2020), before its second, Delta-variant wave (April 2021), and before the country began vaccinating its population in large numbers (March 2021).

### Target population

The study sought to estimate humoral and cellular immunity in two subpopulations of Bangalore, India: residents of slums and residents of non-slums. Slums are defined as communities of squatters without legal rights to the land they occupy, communities sometimes called informal settlements. The choice of strata was motivated by two factors. The first is a hypothesis that slums have higher levels of population density and poverty, both of which might affect disease likelihood, transmission, and impact. Second, a prior survey suggested seroprevalence was several times greater in slums than non-slums (of Mumbai) by July 2020^22^.

### Sample size

The required sample sizes for estimating the positivity rate for a given test within a single community or sample were calculated to conduct a one-sided test with 95% confidence and 80% power and a minimum detectable effect of 10 percentage points (pp). A prior study^23^ found that positivity rates for antibodies to RBD was 52.5% and for RT-PCR was 5.9% in urban Bangalore by August 2020. Assuming that those with infection converted to serological reactivity to SARS-CoV-2 spike, this implies that as many as 58.4% are seropositive after that point. With this expected positivity rate for both humoral and cellular immunity, the required sample size is 191 per community. Our actual sample sizes were greater than this.

The required sample size for estimating the difference in positivity rates for antibodies to the spike protein between two communities or samples, using the same confidence, power, minimum detectable effect parameters, and the same expected positivity rate parameters for each community, is 382 per community. This is also the required sample size for estimating the difference in positivity rates between tests for immunity irrespective of community with the same parameters and expected positivity rates. Our actual sample sizes were greater than this.

### Eligibility criteria

Individuals aged 12 and above were eligible for the study. There were no exclusion criteria.

### Recruitment and sampling

Representative samples in each community type were obtained in four steps. First, 24 starting points in slums and 24 in non-slums across the city of Bangalore were drawn at random with the help of a survey company (Centre for Monitoring Indian Economy). (A map of starting points is in the Supplement.) Second, systematic sampling was employed to recruit individuals for the study. In slums, households live in small, single-family homes. Surveyors approached every 4th home going to the right of the starting point. In non-slum communities, nearly all households live in apartment buildings. Surveyors visited up to 5 buildings: the one closest to the starting point and 4 adjacent buildings to the right. Sampling of households started at the closest building. Surveyors sampled each single-family home selected. In each multi-unit building, surveyors visited 2 households on each floor, specifically the 2nd and 4th to the right of the elevator or stairway. Third, surveyors requested voluntary informed consent to participate in the study from only one person per household. Recruiters used a variant of the Kish method^24^ to recruit members from 8 demographic groups defined by 2 sexes and 4 age categories (12–20, 21–40, 41–60, and > 60). Only individuals who gave informed consent were asked to visit a health camp, which was set up in a central location in each slum or on the sidewalk just outside each non-slum building. The recruitment process was continued until 100 persons consented at each starting point because it was anticipated that only half of the individual subjects who consented would visit the health camp. Fourth, at the health camps, data was gathered from at least the first 50 subjects who visited a camp. More than 50 subjects were sampled if more than 50 arrived at the camp before it closed because the government did not want to turn away volunteers.

### Data and biospecimen collection

Informed consent was obtained from all subjects or the legal guardian(s) of subjects who participated in data or biospecimen collection. Subjects completed a survey and were administered a venous blood draw. The survey included self-reported questions on household demographics, household financial status, and past COVID and vaccination experience. A total of 20ml blood was drawn via two 10ml vacutainers from all the subjects who specifically consented to a blood draw.

### Biospecimen management

After collection, blood samples were refrigerated and sent to Syngene International Ltd. (Bangalore, India) within 4 h. Syngene aliquoted the blood; sent 5 ml of blood to Mapmygenome (Hyderabad, India) for humoral-immunity testing by refrigerated overnight courier; and kept the rest for cellular-immunity testing.

### Humoral immunity tests

Mapmygenome conducted 2 tests for humoral immunity. First, on each sample received, it conducted an ELISA test for IgG antibodies to the S1 spike protein of the SARS-CoV-2 virus using the ELISafe19 kit (HIMEDIA, Mumbai, India, and Syngene). This test has a sensitivity of 99% and specificity of 100%^25^. Second, for each subject whose sample was subject to a cellular immunity test by Syngene, Mapmygenome conducted SARS-CoV-2 Surrogate Virus Neutralization Test (Genscript, Piscataway, NJ, USA), a rapid test for neutralizing antibodies that block the interaction between the receptor binding domain of the spike protein of the SARS-CoV-2 virus with the ACE2 cell surface. This test has 95–100% sensitivity and 99.93% specificity^26^.

### Cellular immunity tests

*PBMC isolation.* Syngene randomly selected 1200 of all blood samples (50% of the first 100 samples taken across sites each day). From these selected samples, it isolated peripheral blood mononuclear cells (PBMCs) from 10ml of blood, created 2 aliquots of PBMCs from each sample, and cryopreserved these aliquots (see Supplement section on PBMC isolation and cryopreservation). Samples that were not selected for PBMC isolation were discarded. From the cryopreserved samples, Syngene further randomly selected one-half (600 specimens) to conduct cellular immunity assays. The remaining PBMC samples were preserved for future studies.

*Peptide synthesis.* The peptide pool used for stimulation of the PBMC consisted of 112 peptides of the spike protein S1, including the RBD, that were 18 amino acids long and overlapping by 12 amino acids. The Supplement provides the characteristics of the peptides. These peptides were synthesized with an average purity of > 90%. The Supplement provides details of peptide synthesis.

*Assays*. From each individual that was selected for a cellular immunity assay, one aliquot was thawed out by swirling the PBMC vial in a 37 °C water bath until just thawed. The thawed PBMCs were added dropwise to a 15ml falcon tube containing 10ml of complete media (RPMI 1640 containing 5% human AB serum and 1% Penicillin/Streptomycin). RPMI 1640 and Penicillin/Streptomycin were purchased from Gibco, USA; human AB serum was purchased from Sigma, USA.) Tubes were centrifuged at 300 × g for 10 min followed by aspiration of the supernatant. Cells were re-suspended in culture medium and plated in 96 well, round-bottom plates at a density of 1 ×  10^6^ cells/well in 150 µl of complete media. To stimulate cytokine release from PBMCs, cells were stimulated only with DMSO controls (0.3% concentration) or also with 200 nM of Spike peptide mix per well for 16h. At the end of 16h, the plates were centrifuged at 1500 rpm for 5 min at 4 °C. Supernatant was collected for secreted cytokine measurements using the Luminex platform. A 10-plex multiplex kit for human cytokines IFN*α*, IFN*β*, IFN*γ*, IL-2, IL-4, IL-6, IL-10, IL-17, TNF*α*, and Granzyme B was custom synthesized from R&D Systems, USA. Each plate included standard curves for IFN*γ* and IL-2 that served as positive control. The negative control was the unstimulated well for each donor. Plates were read on the MAGPIX instrument (Luminex Corp., USA). Standard curves were run for each plate and the data for unknown samples was extrapolated from the standard curves using a 5-parameter non-linear regression curve by the MAGPIX software. Concentration of cytokines was measured in picograms per milliliter (pg/ml).

*Incremental concentration, positivity and positivity rate*. Incremental concentration of a cytokine due to peptide stimulation is defined as concentration of that cytokine in a sample of specimen stimulated with peptides minus the concentration when a sample from the same specimen is stimulated with DMSO controls. A specimen is defined as positive (a binary indicator) for a given cytokine if concentration of that cytokine in stimulated specimen is at least twofold greater than concentration of that cytokine in unstimulated specimen^27–29^. The positivity rate for a given cytokine among a set of specimens is the fraction of those specimens that are positive for the cytokine. When calculating this positivity rate, we do not exclude any specimens that yield samples with high cytokine concentrations with DMSO controls. Average IFN*γ* and IL-2 concentrations for DMSO controls and stimulated samples for all specimens, for specimens with positive incremental concentration, and for specimens with negative incremental concentration for the associated cytokine are reported in Table S3.

To validate our two-fold concentration test for positivity for a cytokine, we estimated a threshold for positivity for a cytokine that equates sensitivity and specificity, a standard approach for determining test cut-off values^30^. To derive this principled threshold, of course, we require knowledge about which samples are truly positive for cellular immunity and which are not, knowledge we do not have. Instead, we assume that if a sample is positive or negative for NAB, it is positive or negative, respectively, for cellular immunity. We then estimate sensitivity (defined as positive for NAB) and specificity (defined as negative for NAB) under different thresholds for the difference in stimulated and stimulated cytokine concentrations. We identify the threshold that gives the same level of sensitivity and specificity. We do this separately for IFN-g and IL-2. As the ROC curve in Figure S.2 shows, the relevant thresholds are 5 for IFN-g and 18.5 for IL-2. These yield a sensitivity and specificity of 65% for each cytokine.

### Outcomes

We have 30 primary outcomes. Twenty of these outcomes, intended to measure immunity levels in different samples, are the positivity rates for 4 tests in 4 samples. The tests are (1) humoral immunity (seroprevalence) measured via (a) ELISA assays for antibodies to the spike protein and (b) rapid tests for neutralizing antibodies (2) cellular immunity measured (a) by IFN-g, (b) by IL-2, and (c) either IFN-g or IL-2 in peptide-stimulated PBMCs. The 4 samples are (1) specimens from slum communities weighted to be demographically representative of those communities, (2) specimens from non-slum communities weighted to be demographically representative of those communities, (3) a sample with specimens from both communities combined, but not weighted at all (“total” samples), and (4) a sample that is representative of the city of Bangalore because it combines slum and non-slum specimens but weights them in proportion to demographics and populations in each community.

The remaining 10 primary outcomes compare immunity across communities and compare immunity implied by different tests. Six outcomes are the differences between slum and non-slum communities in positivity rates for each of the 2 measures of seroprevalence and 3 measures of cellular immunity. Four outcomes compare positivity rates for each of the first 2 measures of cellular immunity (by IFN-g only, by IL-2 only) against positivity rates for each of the 2 humoral immunity tests.

We have 22 secondary outcomes. Twelve outcomes, intended to describe cellular immunity in demographic groups, are positivity rates for cellular immunity measured (a) by IL-2 and (b) by IFN-g production in response to peptide-stimulated PBMCs for each of two sexes and four age groups. Two outcomes, intended to determine if income or density drives immunity rates in slums, are the increment in the partial correlation between slum residence and each measure of cellular immunity when we control for household income.

Our final 8 secondary outcomes, intended to validate the use of IL-2 and IFN-g concentrations as measures of cellular immunity, are (a) concentrations of IL-2 and of IFN-g in pooled samples conditional and unconditional on whether specimens were positive for spike protein (via ELISA) or for neutralizing antibodies and (b) the differences in concentrations of IL-2 and IFN-g between subsamples that are positive and negative for spike protein (via ELISA) or for neutralizing antibodies.

### Statistical analysis

*Positivity for humoral immunity* is defined in either of two ways: a positive test for the spike S1 protein using an ELISA assay or a positive test for neutralizing antibodies (NAB) using a rapid test. There is substantial correspondence between the two tests. Out of 561 specimens for which we had results for both humoral immunity tests, all 88 specimens that were positive for NAB were positive for the spike protein; all tests that were negative for the spike protein, were negative for NAB. The correlation between positivity on the tests was 0.6858. We do not adjust for inaccuracy in the humoral immunity tests by using, for example, the Rogan-Gladen correction^31^, because we are unable to do the same for cellular immunity tests and a central goal is to compare humoral and cellular immunity rates on an apples-to-apples basis.

*Positivity for cellular immunity* is defined in one of three ways. A biospecimen is positive for cellular immunity if it is positive for (1) interferon-gamma (IFN-*γ*), (2) for interleukin-2 (IL-2), or (3) for either IFN-g or IL-2. Positivity rates for cellular immunity are defined as the fraction or percentage of biospecimens for which we have assay results that are positive for cellular immunity. Positivity rates for cellular immunity are calculated from the same individuals as positivity rates for humoral immunity.

We report positivity rates for IFN-*γ* and IL-2 and not other cytokines in our 10-plex kit because these two cytokines have been validated and used for measuring cellular immunity to SARS-CoV-2 and other respiratory pathogens in other studies^2,32–36^. We validate these two cytokines as measures of cellular immunity by comparing the positivity rate or incremental concentration for each cytokine in two subsets of the pooled sample, (a) a subsample that tests positive for spike protein using an ELISA assay or for neutralizing antibodies (NAB) using a rapid test and (b) a subsample that yields a negative result for the same test of humoral immunity. We illustrate the incremental cytokine concentrations by producing box plots showing various percentiles of the incremental concentrations of a cytokine.

The *positivity rates for humoral and cellular immunity in a community type* is estimated in two steps. First, we calculated the positivity rates for each of 8 demographic groups (sex × 4 age groups) in a community using data from members from each group and community that consented to biospecimen collection. Second, we calculate a weighted average of positivity rates for each demographic group in a community. The weights for each demographic group in a community are estimates of the population in each demographic group in each community. The Supplement provides details on calculation of weights.

The *positivity rates for humoral and cellular immunity for a total sample* including both slum and non-slum samples are calculated as the simple fraction of specimens that yield a positive result for the relevant test. Specimens are not weighted before calculating this fraction.

The *positivity rates for humoral and cellular immunity city-wide for Bangalore* are calculated as a weighted average of slum and non-slum positivity rates, with the weights being the population share of slums and non-slums. The urban population share living in slums is 18.58%^37^.

We estimate each of the above positivity rates for the full sample for which we have specimens, as well as for a subsample that excludes donors who report that they were previously vaccinated for SARS-CoV-2.

To estimate the *difference in positivity rates across communities for a given test*, we calculate the difference in weighted-average positivity rates between the two communities. To estimate the *difference in positivity rates across any two tests for immunity* we calculate the difference in unweighted positivity rates between the two tests in a pooled sample of all specimens with a test result.

We estimate *positivity rates for each demographic group* in a pooled sample in two steps. First, we estimate a linear regression of whether a specimen was positive for a given measure of immunity on indicators for each age group and each gender (and no constant) in a pooled sample. Second, we estimate the mean with the coefficient on relevant indicators and a 95% confidence interval using the standard error on the coefficient.

Finally, we measure the *change in the partial correlation coefficient between slum residence and each measure of cellular immunity when we control for income* in two steps. First, we jointly estimate two seemingly unrelated regressions with the pooled sample: one is a linear regression of positivity for cellular immunity on an indicator for slum residence and the other is a regression of positivity for cellular immunity on an indicator for slum residence and subject income in the last month. Second, we use a Wald test to estimate the whether the coefficients on the slum indicator are significantly different.

We employ 2-sided t-tests to determine whether positivity rates, differences in positivity rates or incremental concentrations are different than fixed values or different from each other. Statistical analyses were conducted with Stata 18 (Statacorp)^38^.

## Results

### Sample

Recruiters visited 7,376 homes across 24 sites apiece in slums and in non-slum communities (Table 1). Of the visited homes, households at 8.9% (655) did not answer the door and the selected household member at 29% (2,136) refused consent. Of the 4,585 members that did consent, 3,057 (67%) visited the health camp and were surveyed before the health camp closed for the day.

**Table 1:** Response and consent rate among households (HHs) and selected household members (SHM) during recruitment.

Community	Sites	House-holds visited	Did not answer (HH)	Refused at household (HH)	Consented but did not come to camp (persons)	Consented and came to camp (persons)
Non-slum	24	3,890	366	1,290	740	1,494
Slum	24	3,486	289	846	788	1,563
Total	48	7,376	655	2,136	1,528	3,057
Percent of visited		100	8.9	29.0	20.7	41.4
Percent of consented					33.3	66.7

Table presents the number of households (HH) approached by recruiters by type of community and the response received. The last two columns indicate whether the person selected to participate in the study consented and came to the health camp where biospecimens were taken.

**Figure 1:**
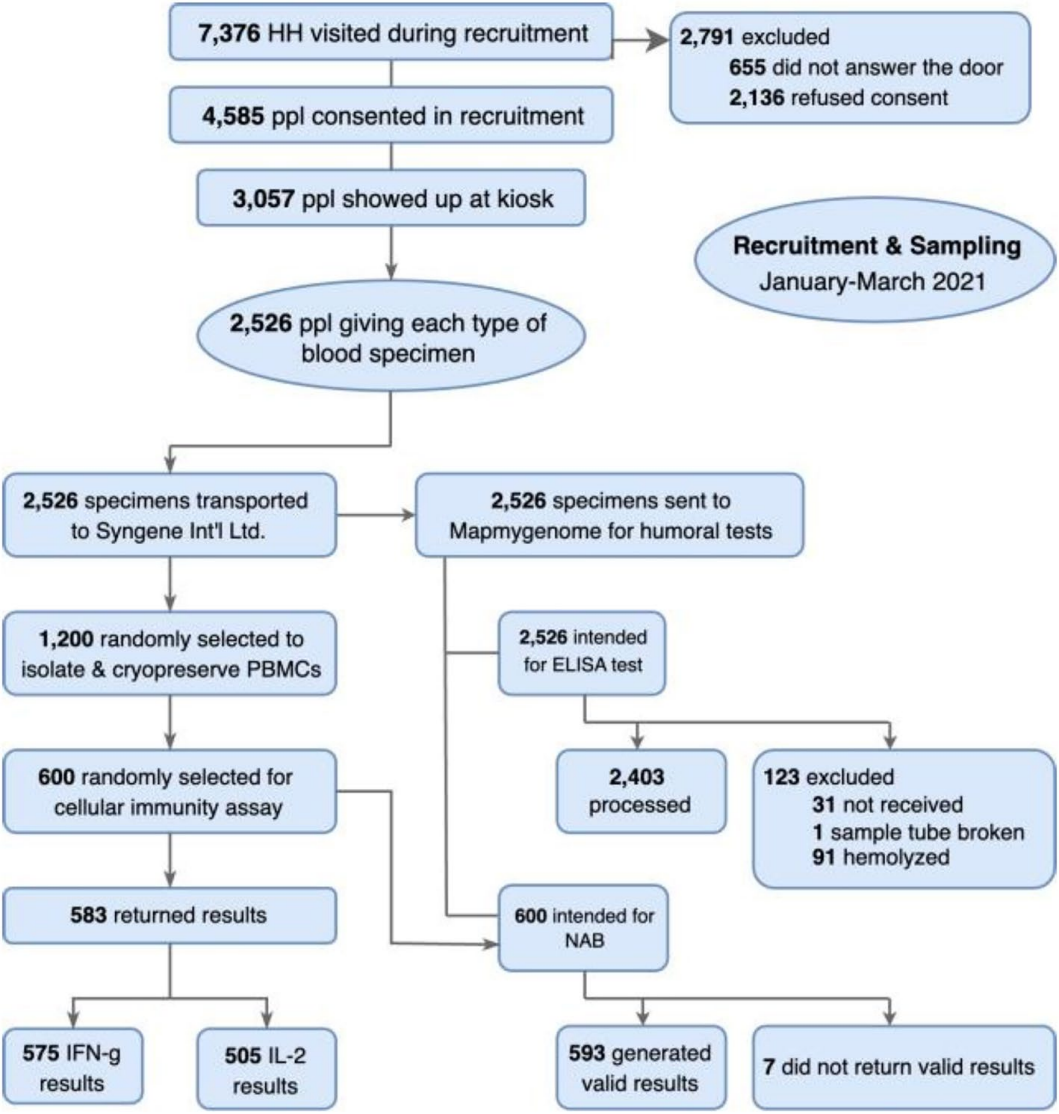
CONSORT diagram.

Of the 3,057 who consented to a verbal survey, 2,526 consented to and provided blood samples (see CONSORT Diagram in Fig. 1). It was intended that all these blood samples would be tested for antibodies to the spike S1 protein (ELISA-spike). However, 123 samples could not be processed because the samples were not received (31), the sample tube was broken (1), or the samples were hemolyzed (91).

Of the 2,526 blood samples, 600 were randomly selected for testing for neutralizing antibodies (NAB) to the RBD of the spike protein and for cellular immunity (CI) assays. However, only 593 returned valid NAB test results. Thawed PBMC aliquots occasionally had inadequate PBMCs, and therefore, some samples did not return CI assay results. A total of 583 samples returned results from CI assays, 575 interferon-gamma (IFN-g) results, and 505 interleukin-2 (IL2) results.

The subjects from slum households were comprised of slightly more females than the overall slum population, and the subjects in both slum and non-slum communities had fewer children than the same-community population (Table 2). The households in slums live in homes with worse infrastructure, higher density, and lower economic status than those from non-slum communities (Table 3). Subjects in slums are 10 pp less likely (p < 0.001) to have a toilet in the home and 27 pp less likely (p < 0.001) to have a water tap. Slum residents have slightly more people in their households (3.99 v. 4.17 in slums, p < 0.001). Respondents from slums on average have 1.3 years less schooling and earn INR 4667 (p < 0.001) and spend INR 3191 (p < 0.001) less per month than those from non-slums. Slum and non-slum communities have similar, low levels of vaccination for SARS-CoV-2: 6.75 and 6.15% (difference p = 0.33).

**Table 2:** Demographics of the sample and population, by type of community.

	Non-slum		Slum	
	Sample	Population	Sample	Population
Female (%)	45.2	46.3	50.4	44.6
Age 12–20	5.0	10.3	4.2	9.4
Age 21–40	20.4	21.1	23.2	20.9
Age 41–60	15.7	12.3	18.6	12.2
Age 61+	4.1	2.5	4.3	2.1
Male (%)	54.8	53.7	49.6	55.4
Age 12–20	6.8	11.1	7.8	12.2
Age 21–40	26.3	24.0	21.5	25.3
Age 41–60	14.6	13.9	14.8	13.9
Age 61+	7.1	4.7	5.6	3.9
Sample size (N)	1494	1494	1563	1563

Sample columns give percent of sample in each demographic category. Population columns give estimates of percent of population in each demographic category. Population estimates are the percent of all members of subjects' households in each category. These population estimates are derived separately from households in each community type.

**Table 3:** Household and individual attributes, by community type.

	Non-slum	Slum	Difference (p)
Home has own toilet	98%	88%	0.00
Home has water tap	100%	77%	0.00
Hours of water/day	6.21	4.24	0.00
Household size (ppl)	3.99	4.17	0.00
Subject employed	51%	52%	0.12
Years of education	9.13	7.75	0.00
Income last month (INR)	20,036	15,369	0.00
Expend. last month (INR)	14,334	11,142	0.00
In debt	46%	49%	0.14
Vaccinated, SARS-Cov-2	6.15%	6.75%	0.33
Sample size (N)	1494	1563	

Non-slum and slum columns give means of items listed in the first column. The last column provides the p-value from a 2-sided t-test of whether the means of each item differ across communities.

### Positivity rates for immunity

Considering all specimens for which we obtain test results, positivity rates for antibodies to spike protein (Table 4), based on ELISA tests are 32 and 31% in slums and non-slums, respectively, both significantly greater than 0 (p < 0.001 each). The difference in serological reactivity was not significant (p = 0.17). The positivity rates for neutralizing antibodies (NAB) are 16 and 12% in slums and non-slums, both significant and significantly different from one another (p < 0.001). The implied Bangalore-wide positivity rates are 31% for ELISA tests and 13% for NAB tests (p < 0.001 each).

**Table 4:** Positivity rates for humoral and cellular immunity, by community type and by sample, as of January–March 2021.

	Slum		Non slum		Unweighted total		Est. Bangalore	
	Pos. Rate	P-value	Pos. Rate	P-value	Pos. Rate	P-value	Pos. Rate	P-value
ELISA	0.321	< 0.001	0.312	< 0.001	0.297	< 0.001	0.314	< 0.001
Obs	1249		1161		481		2410	
NAB	0.163	< 0.001	0.123	< 0.001	0.155	< 0.001	0.131	< 0.001
Obs	328		264		503		592	
IFN-g	0.276	< 0.001	0.282	< 0.001	0.287	< 0.001	0.281	< 0.001
Obs	316		259		506		575	
IL-2	0.409	< 0.001	0.312	< 0.001	0.370	< 0.001	0.329	< 0.001
Obs	275		232		506		507	
IFN-g or IL-2	0.462	< 0.001	0.402	< 0.001	0.460	< 0.001	0.415	< 0.001
Obs	317		259		506		576	

Columns 2–5 give positivity rates for different communities. Rows lists the specific humoral and cellular immunity tests. Pooled sample weights each biospecimen equally. Slums are assumed to be 18.5% of the population of Bangalore when estimating Bangalore-wide cellular immunity levels. "Pos. Rate" column gives fraction of specimens that were positive for a test. "P-value" indicates the p-value from a 2-sided test of statistical significance. Row labeled "Obs." gives number of specimens evaluated in a test.

Using our two-fold concentration test for cytokine positivity, the positivity rates for IFN-g, IL-2 and either IFN-g or IL-2 in unweighted samples were 29, 37 and 46%, respectively.  Positivity rates for IFN-g are 28% both in slums and in non-slums. Positivity rates for IL-2 are 41 and 31% in slums and non-slums. Positivity rates for either cytokine are 46 and 40% in slums and non-slums. The implied positivity rate for production of IFN-g, for IL-2, and for either cytokine Bangalore-wide are 28, 33, and 42%, respectively. Each measure of cellular immunity is significantly different from 0 (p < 0.001) and significantly different across slums and non-slums (p < 0.001). The partial correlation coefficient between slums and cellular immunity is not significantly different whether one controls for subjects’ income in the last month (p > 0.99 each).

Positivity rates for a subsample that excludes vaccinated individuals are < 1 pp different than those for the sample with all possible specimens (Table S4). Using thresholds for cytokine positivity that equate sensitivity and specificity, positivity rates for IFN-g, IL-2 and either IFN-g or IL-2 in unweighted sample are 40, 28, and 46%, respectively.   

### Relationship between humoral and cellular immunity

Cellular immunity is typically more prevalent than humoral immunity. Using incremental concentrations of IFN-*γ* in stimulated PBMC samples as a measure of cellular immunity (Table 4, row 5) and focusing on the unweighted total sample, cellular immunity is 13.2 pp (p < 0.001) greater than humoral immunity measured by neutralizing antibody tests (row 3). Using concentrations of IL-2 to measure cellular immunity (Table 4, row 7), cellular immunity is 7.3 pp (p < 0.001) greater than humoral immunity measured by ELISA tests for IgG antibodies to the spike protein (row 1) and 21.5 pp (p < 0.001) greater than the positivity rate in neutralizing antibody tests. Using concentrations of either cytokine as our measure (Table 4, penultimate row), the respective gaps rise to 16.3 pp and 30.5 pp (p < 0.001).

**Figure 2:**
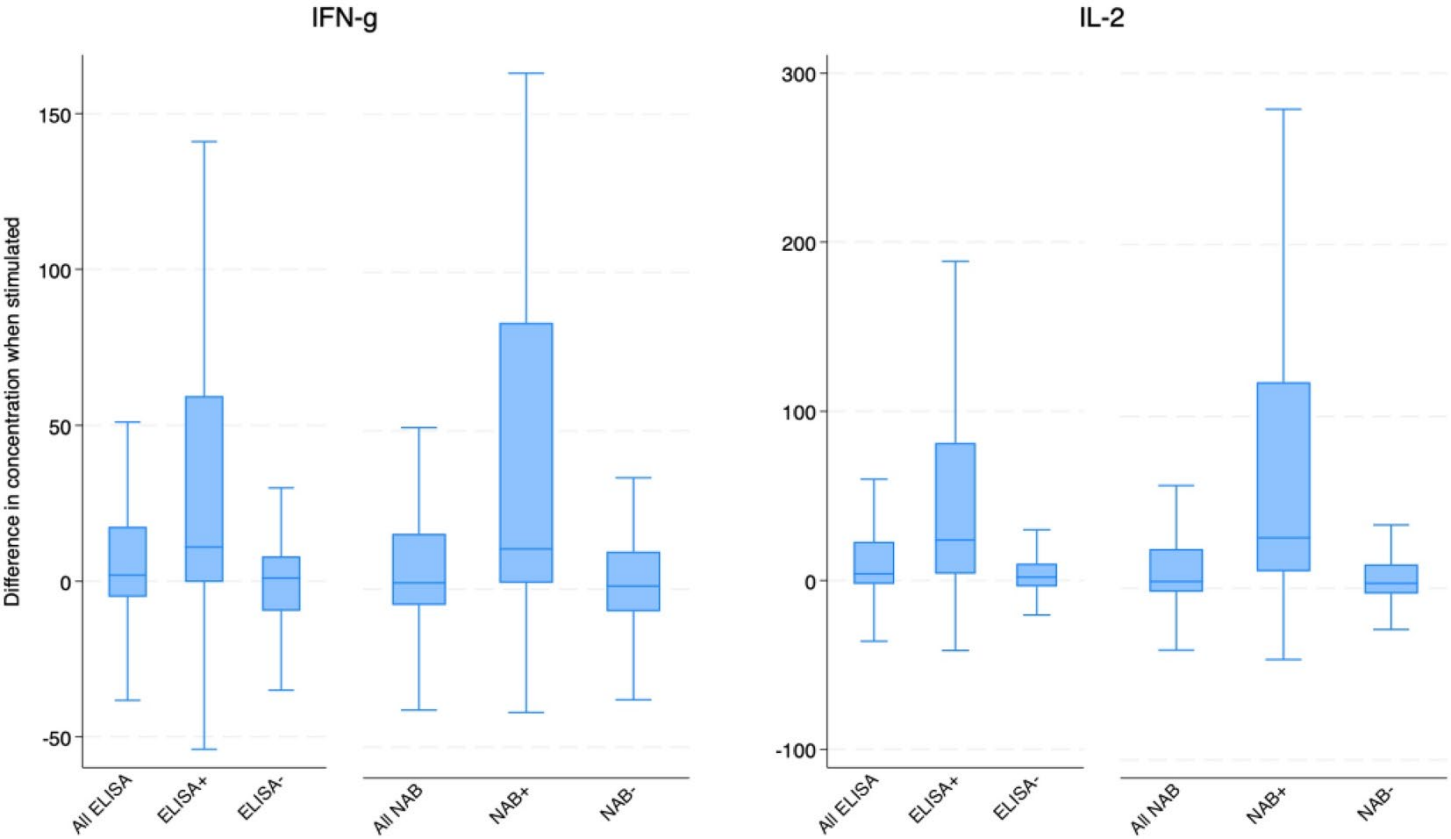
Differences in IFN-g and in IL-2 concentrations when sample is stimulated, by humoral immunity test results. *Note*. Units are pg/ml. Box shows median (with a line) and 25th and 75th percentiles. Whiskers show adjacent values.

**Figure 3:**
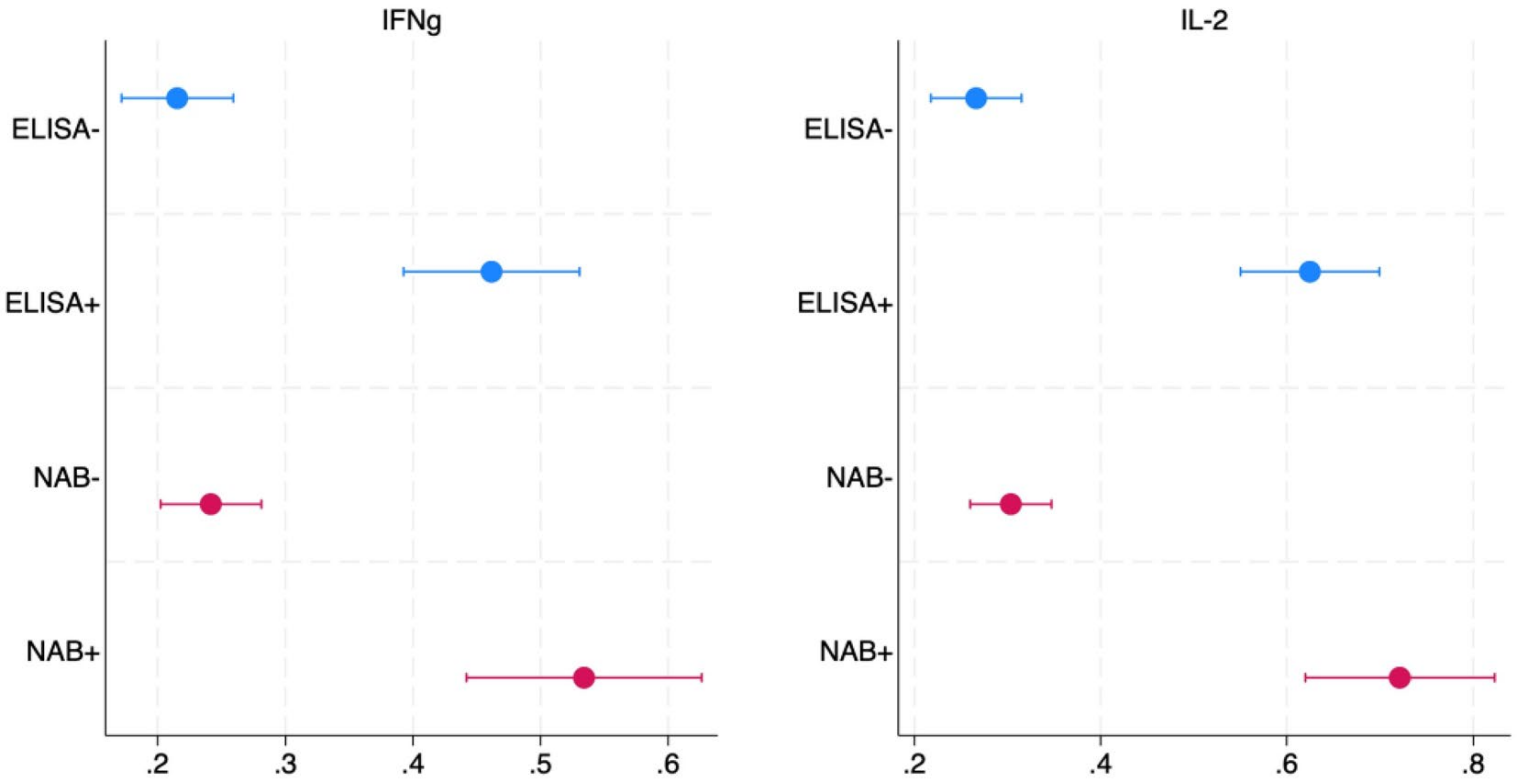
Mean positivity rates and 95% confidence intervals for IFN-g and IL-2 fopr subsamples based on results of humoral immunity tests. *Note*. Dots indicate positivity of cytokine indicated in subplot title and subsample indicated in the row. For example, ELISA+ means subsample that is positive on ELISA and NAB- means negative on nutralizing antibody test. Whiskers indicate 95% confidence intervals for positivity rate for a cytokine assay.

While not all individuals with humoral immunity test positive for cellular immunity based on the threshold we use label an individual’s sample positive for cellular immunity, cellular immunity is more prevalent among those who have humoral immunity. Figure 2 shows IFN-g and IL-2 concentrations, respectively, among subjects who have positive versus negative tests either for IgG antibodies to the spike protein from ELISA tests or for neutralizing antibodies. Median incremental concentrations of both cytokines in samples that are positive for humoral immunity are greater than 75th percentile incremental concentrations in samples that are negative for humoral immunity, however the latter is measured. Mean positivity rates for IFN-g and IL-2 are 23 and 38.7 pp greater (p < 0.001 each), respectively, for those who test positive (versus negative) for antibodies to the spike protein, and 30.8 and 42.3 pp greater (p < 0.001 each), respectively, for those who test positive (versus negative) for neutralizing antibodies (Fig. 3).

### Immunity by age and sex

**Figure 4:**
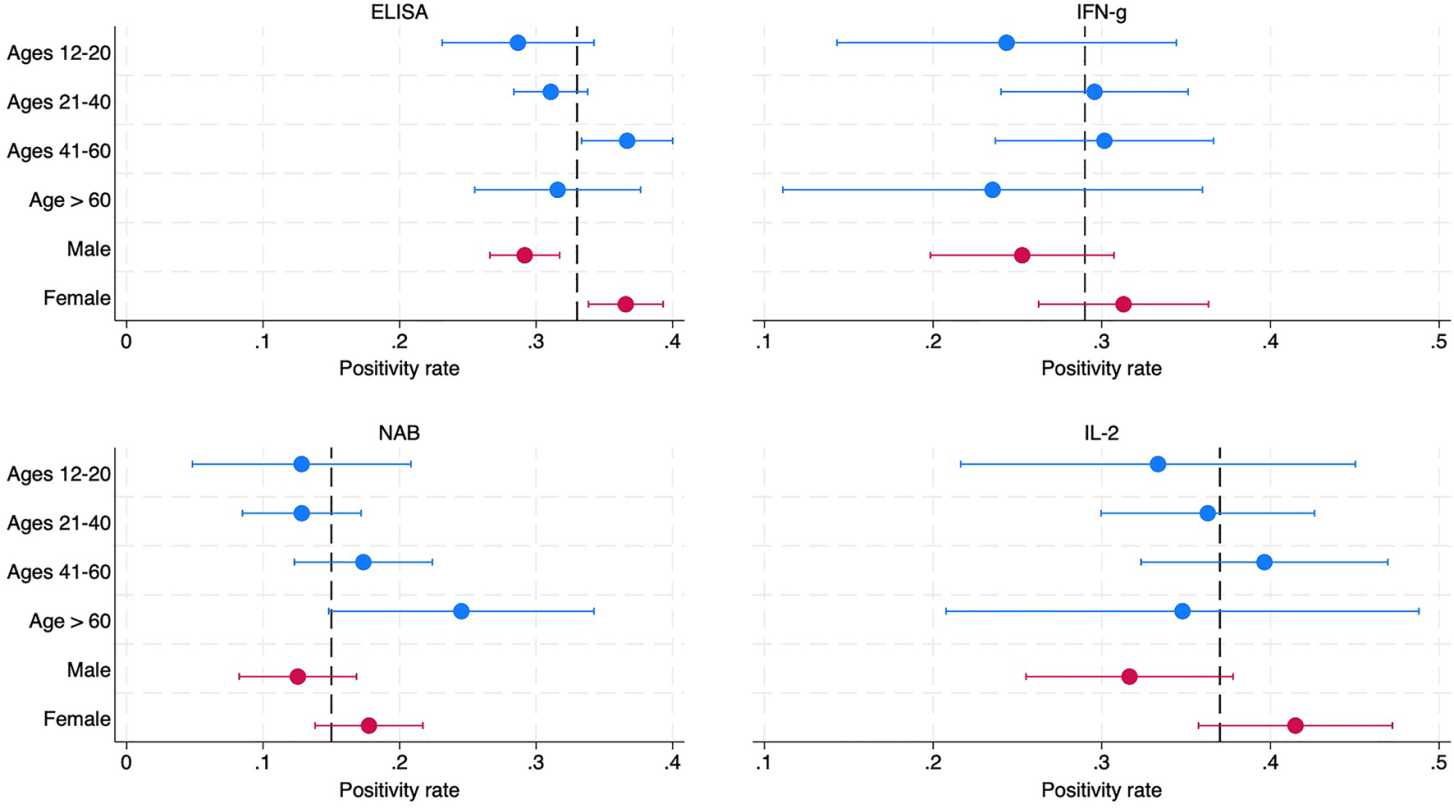
Mean positivity rates and 95% confidence intervals for IFN-g and IL-2 across age and sex. *Note*. Average positivity rates in unweighted sample, indicated by dashed lines, are .33(ELISA), .15(NAB), .29(IFN-g) and .37(IL-2).

Figure 4 presents positivity rates for humoral immunity and cellular immunity for different age groups and sexes. All demographic groups have significant positivity rates (p < 0.001). While positivity rates are typically lower for ages 12–20 (relative to other ages) and always lower for males than females, the difference across age groups are not statistically significant and are significant for sex only for the ELISA test for IgG antibodies to the spike protein (p < 0.001).

## Discussion

This study examines the population-level prevalence of humoral and cellular immunity to SARS-Cov-2 in a large sample from Bangalore, India, from 10 January to 4 March 2021, just before India’s second COVID wave started. The sample size across communities (n = 3057) is larger than the only other population-based sample (n = 2186)^39^. The sample is comprised of two communities (slums and non-slums) allowing us to examine how immunity relates to socio-economic status. The study took place just before the start of India’s vaccination COVID campaign: only ~ 6% of the sample was vaccinated for SARS-CoV-2. Therefore, evidence of immunity in this study is largely driven by infection rather than vaccination.

Estimates of the prevalence of humoral immunity depend on how it is measured. If we pool all specimens, 29.7% of subjects test positive for IgG antibodies to the spike S1 protein (via ELISA tests) that should largely reflect SARS-CoV-2 rather than endemic CoV, while just 15.5% test positive for the neutralizing antibody (NAB) rapid test (Table 4), representing a subset of spike S1-specific antibodies that detect binding to the receptor binding domain of Spike. The differences are statistically significant (p < 0.001).

We measure cellular immunity by concentrations of IFN-*γ* and IL-2 in stimulated specimens of PBMCs, a measure that has been validated and used for measuring cellular immunity to SARS-CoV-2 and other respiratory pathogens in other studies^2,32–36^. We validate these measures by demonstrating that positivity rates for these two cytokines in stimulated PBMCs are 23–42.3 percentage points (pp) higher among those subjects who are also positive for humoral immunity (Fig. 3).

As with humoral immunity, the prevalence of cellular immunity depends on how it is measured. In the pooled sample, 55.7% subjects test positive for cellular immunity if one defines a positive result as greater IFN-g concentration following COVID peptide stimulus, but 66.2% if one defines it as greater IL-2 concentration when stimulated (Table 4).

Cellular immunity was found to be far more prevalent than humoral immunity in most of our tests cellular immunity. Even so, our estimate of cellular immunity is likely an underestimate for two reasons. First, because of the use of a peptide pool that is limited to a subset of the spike protein. While the peptide pool in our assay increases the specificity of T cell reactivity to SARS-CoV-2, it fails to capture COVID-induced T cells specific for other regions of the spike protein. Second, while our assays include a negative control, they do not include a positive control because we lack adequate number of PBMC for each donor to do so. Some of our specimens may have poor viability, which would hinder response to peptide stimulus and cause us to underestimate the proportion of study participants with cellular immunity. Measured just 4 to 5 months after the peak of India’s first wave, our results imply that tests for humoral immunity may substantially underestimate cellular immunity in a rather short period^40^. This finding is consistent the conclusion that either individuals with weak humoral immunity response to SARS-CoV-2 may generate cellular immunity^41^ or that humoral immunity to SARS-CoV-2 is short-lived^4,41,42^.

While demographics (age or sex) do not predict significant differences in cellular immunity, residence in a slum certainly does. Subjects living in slums have 9.7 pp higher positivity rate (p < 0.001) for cellular immunity than those in non-slums (who have 31.2% positivity), when cellular immunity is measured by positivity for IL-2. It is likely the correlation between cellular immunity and slum residence is mediated by population density rather than income as the partial correlation of cellular immunity and residence in slums and cellular immunity does not fall significantly when we control for income. Finally, humoral immunity rates are significantly greater in slums than non-slums when measured via NAB test, but not when measured via ELISA test for IgG antibodies to the spike protein.

The study has at least two limitations related to the experiments performed. First, because of the number of samples analyzed, and the relatively small blood volumes collected, our measurements did not distinguish the frequency of SARS-CoV-2 reactive CD4 and CD8 T cells, but rather the accumulated cytokines produced in overnight culture of antigen-stimulated PBMC. Second, we have estimated SARS-CoV-2 cellular immunity based on reactivity to a segment of spike protein and did not measure the additional responses to nucleocapsid and membrane, which are known to be targets of T cells^2,43^ and thus our conclusions regarding the frequencies of cellular immunity may be underestimates. Despite these limitations, this study suggests that population-level humoral surveys may underestimate total immunity, which include cellular immunity. It also demonstrated that population-level cellular immunity surveys are feasible, even in a low-income setting during a pandemic.

### Supplementary Information

Supplementary Information.

## Data Availability

De-identified versions of the datasets used and/or analysed during the current study available from the corresponding author on reasonable request.
