# Supplementary material for: Comparing population-level humoral and cellular immunity to SARS-Cov-2 in Bangalore, India
Source: Sci Rep. 2024 Mar 8;14:5758. doi: 10.1038/s41598-024-54922-z (PMC10923858; doi:10.1038/s41598-024-54922-z)
Supplement: Supplementary file 1 — Supplementary Information. [file 41598_2024_54922_MOESM1_ESM.docx]

SUPPLEMENTAL MATERIALS

Comparing Population-Level Humoral and

Cellular Immunity to SARS-Cov-2 in Bangalore, India

Anup Malani, Jayashree Aiyar, Andrea Sant, Neha Kamran,

Manoj Mohanan, Saloni Taneja, Bartek Woda, Wanran Zhao, and Anu Acharya

# Methods

**Additional information on approvals**. This study was approved by the Government of India (the Prime Minister’s Principal Scientific Advisor’s Office) and the Government of Karnataka (Commissionerate Health & Family Welfare, No. NVBDCP/ EST 7F/COVID-19/ 2020-21). The study protocols were also approved by IRB / IEC committees at two institutions:

- Karesa (ECR/308/Indt/KA/2018) IRB, approved February 11, 2021, for Anu Acharya (Mapmygenome),
- University of Chicago (IRB20-1484).

Because Malani (University of Chicago) only received de-identified data, the research was determined to be exempt from IRB review at their institutions.

Informed consent was requested at three points: when recruiting individuals, when individuals reported to health camps, and just before blood was taken. The first consent concerned the recruitment questionnaire, the second the oral survey and anthropometric measurements taken at the health camp, and the third the venous blood draw. Data was only gathered from an individual if they consented.

**Sampling**. The 48 starting points (Figure S1) were visited in a sequence that would minimize the duration of biospecimen collection. Each day 2 starting points were visited, one in a slum and one in the closest non-slum community. The route through slum (and thus non-slum) sites was selected at random.

## Figure S1. Map of slum and non-slum starting points for systematic sampling.


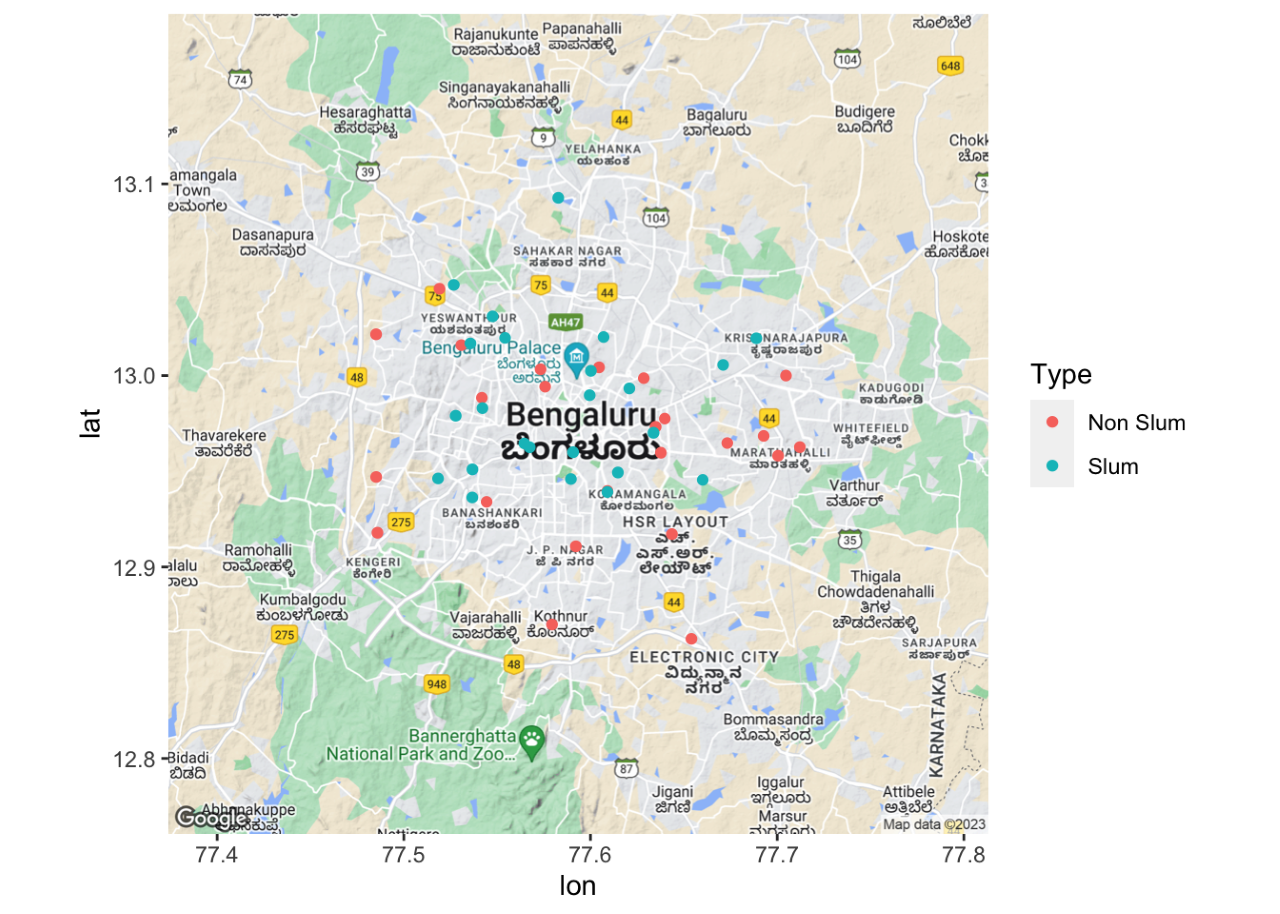


Notes. Map of Bangalore, excluding slum and non-slum study locations, © 2021 Google ^1^.

Each starting point is visited just one day. If less than 50 persons provide biospecimens at a starting point, the deficit (50 - number providing specimens) is allocated to remaining starting points from the same type of community that have not yet been visited. If, during systematic sampling in a slum, a household does not answer the door, it is replaced with the adjacent household to the right. We selected an individual to sample within the household using the following Table S1:

## Table S1. Demographic groups for employing Kish method.

| Male, 12-20 | Female, 12-20 |
| --- | --- |
| Male, 21-40 | Female, 21-40 |
| Male, 41-60 | Female, 41-60 |
| Male, 61+ | Female, 61+ |

At the 1st household surveyors asked a person from the first demographic cell, 2nd household a person from the second cell, and so on. If the designated person is not available, surveyors asked for consent from someone in the next cell. If, as a result, a surveyor skipped a cell in one household, in the next household the surveyor would start with the skipped cell before going to the next open cell. If the designated person refuses consent, the surveyor skips to the next household and asks for someone in the same cell. The goal is to sample 2 persons for each cell over the course of every 16 households.

**Service providers**. The listing exercise, oral survey, and biospecimen collection was conducted by Morsel. The municipal government in Bangalore provided ASHA workers who were known to local residents to accompany surveyors conducting the listing exercise to encourage participation. The ELISA test, PBMC isolation, and the test for cellular immunity was conducted by Syngene International LTD. The rapid neutralizing antibody test was conducted by Mapmygenome India LTD.

**PBMC isolation and cryo-preservation**. Venous blood was collected in sodium heparin tubes at the site of clinical examination. Peripheral blood mono-nuclear cells (PBMCs) were isolated from whole blood samples by density centrifugation with Lymphoprep (STEMCELL Technologies). Briefly, 10 mL Lymphoprep was added to falcon tubes. Blood was diluted with an equal amount of Roswell Park Memorial Institute (RPMI) 1640 medium (Gibco) + 1% PenStrep (Gibco). Diluted blood was gently layered on top of Lymphoprep. Tubes were centrifuged at 430 RCF for 30 minutes at room temperature. Post centrifugation, a buffy coat of PBMC layer was transferred into a fresh tube without disturbing the erythrocyte/granulocyte pellet. Pellet was washed twice with DPBS (Gibco) and RBCs were removed by using the ACK lysis buffer (Gibco). Post RBC lysis, the pellet was washed and resuspended in RPMI 1640 media with 10% FBS (Gibco) and cells were counted. Isolated PBMCs were cryo-preserved in a freezing solution containing 90% FBS and 10% dimethyl sulfoxide (Sigma) and stored in liquid nitrogen until use.

**Peptide synthesis**. Peptide synthesis was carried out using pre-loaded Wang resin (0.4 mmol/g, 0.08 mmol scale) on an automated Syro-1 parallel peptide synthesizer by Fmoc-SPPS/tBu strategy. Double coupling of Fmoc-protected amino acids (0.3 mol) was performed by using DIC (0.3 mol)/Oxyma (0.3 mol) for 40 min, and HATU (0.29 mol)/DIPEA (0.6 mol) for 30 min successively. The resin was washed with DMF (3 x 1 min) after each coupling. Fmoc-deprotection was accomplished with 40% piperidine for 3 min and 20% piperidine for 12 min. After Fmoc-deprotection, the resin was washed with DMF (6 x 1 min). Finally, the resin was drained and washed with DMF, DCM, and Diethyl ether. The resulting peptidyl resin was cleaved with either TFA/TIS/H2O (95:2.5:2.5, v/v/v) or TFA/TIS/DODT/H2O (92.5:2.5:2.5:2.5, v/v/v/v) for 3 h. The peptide was precipitated in cold ether and collected by centrifugation. The crude peptide was purified by a preparative reverse-phase HPLC with 0.1 % TFA in H2O/ACN as gradient. Fractions were collected and lyophilized to yield the peptides used for T cell stimulation.

**Peptides**. Table S2 characterizes the S1 peptides synthesized for this study.

## Table S2. Sequence for synthesized S1 peptides.

| No | Sequence | No | Sequence | No | Sequence | No | Sequence |
| --- | --- | --- | --- | --- | --- | --- | --- |
| **1** | **MFVFLVLLPLVSSQCVNL** | **31** | **GKQGNFKNLREFVFKNID** | **61** | **CVADYSVLYNSASFSTFK** | **91** | **FNFNGLTGTGVLTESNKK** |
| **2** | **LLPLVSSQCVNLTTRTQL** | **32** | **KNLREFVFKNIDGYFKIY** | **62** | **VLYNSASFSTFKCYGVSP** | **92** | **TGTGVLTESNKKFLPFQQ** |
| **3** | **SQCVNLTTRTQLPPAYTN** | **33** | **VFKNIDGYFKIYSKHTPI** | **63** | **SFSTFKCYGVSPTKLNDL** | **93** | **TESNKKFLPFQQFGRDIA** |
| **4** | **TTRTQLPPAYTNSFTRGV** | **34** | **GYFKIYSKHTPINLVRDL** | **64** | **CYGVSPTKLNDLCFTNVY** | **94** | **FLPFQQFGRDIADTTDAV** |
| **5** | **PPAYTNSFTRGVYYPDKV** | **35** | **SKHTPINLVRDLPQGFSA** | **65** | **TKLNDLCFTNVYADSFVI** | **95** | **FGRDIADTTDAVRDPQTL** |
| **6** | **SFTRGVYYPDKVFRSSVL** | **36** | **NLVRDLPQGFSALEPLVD** | **66** | **CFTNVYADSFVIRGDEVR** | **96** | **DTTDAVRDPQTLEILDIT** |
| **7** | **YYPDKVFRSSVLHSTQDL** | **37** | **PQGFSALEPLVDLPIGIN** | **67** | **ADSFVIRGDEVRQIAPGQ** | **97** | **RDPQTLEILDITPCSFGG** |
| **8** | **FRSSVLHSTQDLFLPFFS** | **38** | **LEPLVDLPIGINITRFQT** | **68** | **RGDEVRQIAPGQTGKIAD** | **98** | **EILDITPCSFGGVSVITP** |
| **9** | **HSTQDLFLPFFSNVTWFH** | **39** | **LPIGINITRFQTLLALHR** | **69** | **QIAPGQTGKIADYNYKLP** | **99** | **PCSFGGVSVITPGTNTSN** |
| **10** | **FLPFFSNVTWFHAIHVSG** | **40** | **ITRFQTLLALHRSYLTPG** | **70** | **TGKIADYNYKLPDDFTGC** | **100** | **VSVITPGTNTSNQVAVLY** |
| **11** | **NVTWFHAIHVSGTNGTKR** | **41** | **LLALHRSYLTPGDSSSGW** | **71** | **YNYKLPDDFTGCVIAWNS** | **101** | **GTNTSNQVAVLYQDVNCT** |
| **12** | **AIHVSGTNGTKRFDNPVL** | **42** | **SYLTPGDSSSGWTAGAAA** | **72** | **DDFTGCVIAWNSNNLDSK** | **102** | **QVAVLYQDVNCTEVPVAI** |
| **13** | **TNGTKRFDNPVLPFNDGV** | **43** | **DSSSGWTAGAAAYYVGYL** | **73** | **VIAWNSNNLDSKVGGNYN** | **103** | **QDVNCTEVPVAIHADQLT** |
| **14** | **FDNPVLPFNDGVYFASTE** | **44** | **TAGAAAYYVGYLQPRTFL** | **74** | **NNLDSKVGGNYNYLYRLF** | **104** | **EVPVAIHADQLTPTWRVY** |
| **15** | **PFNDGVYFASTEKSNIIR** | **45** | **YYVGYLQPRTFLLKYNEN** | **75** | **VGGNYNYLYRLFRKSNLK** | **105** | **HADQLTPTWRVYSTGSNV** |
| **16** | **YFASTEKSNIIRGWIFGT** | **46** | **QPRTFLLKYNENGTITDA** | **76** | **YLYRLFRKSNLKPFERDI** | **106** | **PTWRVYSTGSNVFQTRAG** |
| **17** | **KSNIIRGWIFGTTLDSKT** | **47** | **LKYNENGTITDAVDCALD** | **77** | **RKSNLKPFERDISTEIYQ** | **107** | **STGSNVFQTRAGCLIGAE** |
| **18** | **GWIFGTTLDSKTQSLLIV** | **48** | **GTITDAVDCALDPLSETK** | **78** | **PFERDISTEIYQAGSTPC** | **108** | **FQTRAGCLIGAEHVNNSY** |
| **19** | **TLDSKTQSLLIVNNATNV** | **49** | **VDCALDPLSETKCTLKSF** | **79** | **STEIYQAGSTPCNGVEGF** | **109** | **CLIGAEHVNNSYECDIPI** |
| **20** | **QSLLIVNNATNVVIKVCE** | **50** | **PLSETKCTLKSFTVEKGI** | **80** | **AGSTPCNGVEGFNCYFPL** | **110** | **HVNNSYECDIPIGAGICA** |
| **21** | **NNATNVVIKVCEFQFCND** | **51** | **CTLKSFTVEKGIYQTSNF** | **81** | **NGVEGFNCYFPLQSYGFQ** | **111** | **ECDIPIGAGICASYQTQT** |
| **22** | **VIKVCEFQFCNDPFLGVY** | **52** | **TVEKGIYQTSNFRVQPTE** | **82** | **NCYFPLQSYGFQPTNGVG** | **112** | **GAGICASYQTQTNSPRRA** |
| **23** | **FQFCNDPFLGVYYHKNNK** | **53** | **YQTSNFRVQPTESIVRFP** | **83** | **QSYGFQPTNGVGYQPYRV** | **113** | **SYQTQTNSPRRARSVASQ** |
| **24** | **PFLGVYYHKNNKSWMESE** | **54** | **RVQPTESIVRFPNITNLC** | **84** | **PTNGVGYQPYRVVVLSFE** | **114** | **NSPRRARSVASQSII** |
| **25** | **YHKNNKSWMESEFRVYSS** | **55** | **SIVRFPNITNLCPFGEVF** | **85** | **YQPYRVVVLSFELLHAPA** | **115** | **TQTNSPRRARSVASQSII** |
| **26** | **SWMESEFRVYSSANNCTF** | **56** | **NITNLCPFGEVFNATRFA** | **86** | **VVLSFELLHAPATVCGPK** |  |  |
| **27** | **FRVYSSANNCTFEYVSQP** | **57** | **PFGEVFNATRFASVYAWN** | **87** | **LLHAPATVCGPKKSTNLV** |  |  |
| **28** | **ANNCTFEYVSQPFLMDLE** | **58** | **NATRFASVYAWNRKRISN** | **88** | **TVCGPKKSTNLVKNKCVN** |  |  |
| **29** | **EYVSQPFLMDLEGKQGNF** | **59** | **SVYAWNRKRISNCVADYS** | **89** | **KSTNLVKNKCVNFNFNGL** |  |  |
| **30** | **FLMDLEGKQGNFKNLREF** | **60** | **RKRISNCVADYSVLYNSA** | **90** | **KNKCVNFNFNGLTGTGVL** |  |  |

**Calculation of weighted averages**. The recruitment survey asked the individual selected as a respondent in each household to give not only their age and sex, but also the age and sex of every other household member. The respondent’s demographics are used to estimate the fraction of the population from each community that is each of the 8 demographic groups from which we sampled. Specifically, our estimate of the fraction of people in demographic group j (from Table S1) in community type k (slum or non-slum) is

$$F_{jk}=(1/m_{k})\sum_{i=1}^{n_{k}} m_{ijk}$$

where $m_{ijk}$ is the number of household members in person i’s household in demographic group j in community type k, $n_{k}$ is the number of respondents who consented during recruitment in community type k, and

$$m_{k}=\sum_{j=1}^{8} \sum_{i=1}^{n_{k}} m_{ijk}$$

is the number of members in the households of all recruitment respondents in community type k.

Let $y_{ijk}$ be the outcome of a test for member i of the biospecimen sample from demographic group j in community type k. Then the estimate of the community-type average outcome is

$$y_{k}=\sum_{j=1}^{8} F_{jk}\left[ \left( \frac{1}{n_{jk}} \right)\sum_{i=1}^{n_{k}} d_{ijk}y \right]$$

where $n_{jk}$ is the number of biospecimen sample members in demographic group j in community type k.

# Results

## Table S3. Concentration of cytokines in control specimen and incremental concentration of cytokines in stimulated specimen, conditional on whether incremental concentration in stimulated specimens is positive.

|  | **IFN-****γ** | | |  | **IL -2** | | |
| --- | --- | --- | --- | --- | --- | --- | --- |
|  | **All specimens** | **Specimens with non-positive incr. conc.** | **Specimens with positive incr. conc.** |  | **All specimens** | **Specimens with non-positive incr. conc.** | **Specimens with positive incr. conc.** |
| **DMSO control** | 181.528 | 235.269 | 139.895 |  | 16.917 | 20.719 | 14.965 |
| **(conc.)** | (31.129) | (53.409) | (36.515) |  | (1.029) | (1.965) | (1.175) |
| **Specimen** | 63.074 | -156.699 | 233.330 |  | 37.679 | -8.491 | 61.385 |
| **(incr. conc.)** | (38.504) | (43.312) | (57.839) |  | (6.827) | (1.010) | (10.081) |
| Notes. Units are pg/ml. Cells contain means and standard errors (in parentheses) of concentrations of values indicated in the rows headings in samples indicated column headings. Concentration is abbreviated "conc." and incremental "incr.". Specimens may have non-positive incremental concentration of a cytokine if the unstimulated wells of the specimen have cytokine concentrations that are at least as high as the cytokine concentrations in stimulated wells of the specimen. | | | | | | | |

## Table S4: Positivity rates for humoral and cellular immunity, by community type and by sample, as of January - March 2021, excluding donors who were vaccinated for SARS-CoV-2.

|  |  | **Slum** | |  | **Non Slum** | |  | **Unweighted Total** | |  | **Est. Bangalore** | |
| --- | --- | --- | --- | --- | --- | --- | --- | --- | --- | --- | --- | --- |
|  |  | **Pos. Rate** | **P-value** |  | **Pos. Rate** | **P-value** |  | **Pos. Rate** | **P-value** |  | **Pos. Rate** | **P-value** |
| **ELISA** | | 0.317 | 0.000 |  | 0.311 | 0.000 |  | 0.297 | 0.000 |  | 0.312 | 0.000 |
|  | Obs. | 1162 |  |  | 1088 |  |  | 451 |  |  | 2250 |  |
| **NAB** | | 0.163 | 0.000 |  | 0.120 | 0.000 |  | 0.150 | 0.000 |  | 0.128 | 0.000 |
|  | Obs. | 314 |  |  | 243 |  |  | 472 |  |  | 557 |  |
| **IFN-g** | | 0.271 | 0.000 |  | 0.291 | 0.000 |  | 0.288 | 0.000 |  | 0.287 | 0.000 |
|  | Obs. | 302 |  |  | 238 |  |  | 475 |  |  | 540 |  |
| **IL-2** | | 0.409 | 0.000 |  | 0.312 | 0.000 |  | 0.371 | 0.000 |  | 0.330 | 0.000 |
|  | Obs. | 263 |  |  | 213 |  |  | 475 |  |  | 476 |  |
| **IFN-g or IL-2** | | 0.462 | 0.000 |  | 0.408 | 0.000 |  | 0.463 | 0.000 |  | 0.420 | 0.000 |
|  | Obs. | 303 |  |  | 238 |  |  | 475 |  |  | 541 |  |
| Notes. Sample for this table excludes individuals who report that they were previous vaccinated for SARS-CoV-2. Columns 2-5 give positivity rates for different communities. Rows lists the specific humoral and cellular immunity tests. Pooled sample weights each biospecimen equally. Slums are assumed to be 18.5% of the population of Bangalore when estimating Bangalore-wide cellular immunity levels. "Pos. Rate" column gives fraction of specimens that were positive for a test. "P-value" indicates the p-value from a 2-sided test of statistical significance. Row labeled "Obs." gives number of specimens evaluated in a test. | | | | | | | | | | | | |

## Figure S2. Receiver operating characteristic (ROC) curves for different thresholds for IFN- $\gamma$ and IL-2 positivity, assuming neutralizing antibody (NAB) exactly measures cellular immunity, and thresholds for cytokine positivity that equate sensitivity and specificity for cellular immunity.

*Notes. This figure presents the sensitivity (Se, on y-axis) and specificity (Sp, on x-axis) of different thresholds for positivity for a cytokine (IFN-*$\gamma$ *or IL-2). First, for each donor, we calculate the difference between cytokine concentration (pg/ml) in stimulated and unstimulated specimens of PBMC from that donor. For a given numerical threshold, a donor is defined for a cytokine if their difference is greater than that threshold, and negative otherwise. Sensitivity of a given threshold for a cytokine is defined as the fraction of (a) donors that test positive for neutralizing antibodies (NAB) that (b) test positive for a cytokine at that threshold. Specificity of a given threshold for a cytokine is defined as the fraction of (a) donors that test negative for NAB that (b) test negative for a cytokine at that threshold. The blue (IFN-*$\gamma$*) and red (IL-2) lines plot the sensitivity and false positive rate (defined as 1 – specificity) combinations for a range of thresholds. In general, greater sensitivity implies lower specificity. The dashed blue and red lines show the sensitivity (x-axis) for thresholds (5 pg/ml and 18.5 pg/ml) that equate sensitivity and specificity for IFN-*$\gamma$ *and IL-2, respectively.*

# References

1 Google. *[Map of Bangalore], retrieved January 9, 2021, from maps.google.com*, (n.d.).
